# Characterization of features and outcomes of young patients (< 45 years) presenting with ST-segment elevation myocardial infarction

**DOI:** 10.1186/s43044-023-00357-2

**Published:** 2023-04-25

**Authors:** Ahmad Samir, Mohammed Almahjori, Basem Zarif, Mai Elshinawi, Hesham Yehia, Mohamed Elhafy, Ahmed Shehata, Azza Farrag

**Affiliations:** 1 Faculty of Medicine, Cairo University, Cairo, Egypt; 2 National Heart Institute, Cairo, Egypt

**Keywords:** ST-segment elevation myocardial infarction (STEMI), Young ≤ 45 years—premature coronary artery disease (CAD), Egypt

## Abstract

**Background:**

Coronary artery disease (CAD) is the commonest cause of death worldwide. ST-segment elevation myocardial infarction (STEMI) and its consequences can be devastating particularly at younger age for a bigger impact on the patient’s psychology and ability to work. Little is known about the differential features and outcomes of young STEMI patients in Egypt. This study characterized young STEMI patients (≤ 45 years) compared to patients > 45 years and evaluated 1-year outcomes.

**Results:**

A total of 492 eligible STEMI patients who presented to the National Heart Institute and Cairo University Hospitals were recruited. Young STEMI patients (< 45 years old) represented 20% of all STEMI comers. Male gender was predominant in both groups, yet with a significantly higher proportion in the younger compared to older patients (87% vs. 73%, *p* = 0.004). Compared to older patients, young STEMI patients had characteristically higher rates of smoking (72.4% vs. 49.7%, *p* < 0.001) and family history (13.3% vs. 4.8%, *p* = 0.002), while significantly lower rate of other conventional CAD risk factors as diabetes, hypertension, and dyslipidemia (20.4% vs. 44.7%, 20.4% vs. 44.9% and 12.7% vs. 21.8%, respectively, *p* < 0.05 for all). Follow-up was continued for at least 12 months after the index event. Younger STEMI patients had fewer major adverse cardiovascular events and fewer heart failure hospitalizations compared to the older controls (10.2 vs. 23.9% and 18.4% vs. 34.8%, respectively, *p* < 0.005 for both), however, 1-year mortality was similar (3.1% vs. 4.1%, *p* = 0.64).

**Conclusions:**

Younger STEMI patients (≤ 45 years) show peculiar characteristics, with significantly higher rates of smoking and family history of premature CAD, while less prevalence of other conventional CAD risk factors. Overall MACE occurred less in younger STEMI patients; however, the mortality rate was similar to the older controls.

## Background

Coronary artery disease (CAD) is the leading cause of death worldwide [1]. Despite the huge advance in the current understanding and management of CAD, literature focusing on premature CAD and acute myocardial infarction (AMI) in the young is lacking [2]. Consequences of ST-segment elevation myocardial infarction (STEMI) can be devastating particularly when occurs at young age for the bigger impact on the patient’s psychology, ability to work and the extended socioeconomic and health burdens. Also, young STEMI patients are often the primary family earners, hence, the aftermath of STEMI usually affects multiple dependents as well as the national productivity [2].

There is a disparity in the literature on the definition of young concerning premature CAD and myocardial infarction (MI). The term young varies from ≤ 40, ≤ 45 or ≤ 55 years of age 3–5]. Although it seems that ≤ 45 years is the most accepted cut-off when defining young in respect to acute MI [5], although family history of premature CAD often refers to MI ≤ 55 years or ≤ 65 years for male and female relatives, respectively [6, 7]. Besides the unsettled age definition, MI at a young age remains a significant problem, particularly in developing countries and the Middle East, that was not adequately investigated.

The INTERHEART Study reported that the Middle East had the youngest median age for the first MI (51 years), approximately a decade younger than North America (59 years) and Western Europe (63 years) [8]. Middle-East and North Africa (MENA) countries had the highest proportion of individuals ≤ 40 years with MI at 11%, contrasted to 4% in North America and 3% in Western Europe. These findings flag a substantial alarm for MENA countries as the rising rates of premature MI can hugely impact the national productivity and the extended healthcare expenditures with subsequent overburdening of their developing economies [8].

This study targeted evaluation of the risk factors, predictors and differential outcomes in young patients (≤ 45 years) contrasted to the older controls in an Egyptian STEMI cohort, aiming to recognize potential corrective strategies for this critical group.

## Methods

This prospective cross-sectional study targeted evaluation of the risk factors, predictors and differential outcomes in young patients (≤ 45 years) contrasted to the older controls in an Egyptian STEMI cohort, aiming to recognize potential corrective strategies for this critical group. This study recruited all STEMI patients presenting for primary PCI (pPCI) in two large Egyptian referral centers, National Heart Institute and Cairo University Hospitals, from October 2020 to September 2021. The study protocol was approved by the research ethics committee in both participating centers (MD-287-2020). All of the study cohort provided written informed consent for participation and approved the analysis of their anonymized clinical data. Clinical (patient-related) and angiographic (lesion-related) features of young STEMI patients (≤ 45 years) were contrasted to the older control (> 45 years). Subsequently, the whole study group was followed up for at least 12 months after the index event to evaluate clinical outcomes particularly major adverse cardiovascular events (MACE). MACE was defined as death, non-fatal MI, and target vessel revascularization (TLR) [9]. Inclusion criterion was acute STEMI undergoing pPCI. Exclusion criteria were patient's refusal to participate, inability to provide consent (like patients in coma or impaired cognition), cardiogenic shock and severe renal impairment. STEMI diagnosis was based on the 4th universal definition of myocardial infarction [10]. Cardiogenic shock was defined as presentation with signs of impaired peripheral perfusion and profound hypotension (90/60 mmHg) necessitating pharmacological or mechanical circulatory support [11]. Severe renal impairment was defined as estimated glomerular filtration rate (eGFR) < 30 ml/min/1.73 m^2^ calculated by MDRD-4 Equation [12].

Study participants were subjected to detailed history-taking emphasizing on age, sex, conventional risk factors for atherosclerotic cardiovascular disease (ASCVD as smoking, hypertension, diabetes mellitus, dyslipidemia and family history of premature CAD. History of any previous ASCVD events (MI, stroke, aortic aneurysm, any revascularization) was sought and detailed. Physical examination during admission focused on vital signs, patients’ weight and height, chest and cardiac auscultations, defining KILLIP class and eliciting any signs of ASCVD (signs suggesting peripheral arterial disease or prior stroke).

Standard 12-lead surface electrocardiogram was obtained followed by a quick echocardiogram to assess left ventricular ejection fraction (LVEF), regional wall motion score (RWMS), wall motion score index (WMSI) and any mechanical complication. Blood samples were withdrawn for routine laboratory work-up including cardiac biomarkers, complete blood count, liver and kidney functions, random blood sugar and detailed lipid profile. Laboratory work-up was evaluated and was used to reclassify patients as diabetic, or dyslipidemic when not previously known, (i.e., first diagnosed). Worth to mention that patients proceeded to pPCI without awaiting laboratory results prioritizing acceleration of coronary reperfusion.

Coronary angiography and pPCI were performed according to standard protocols. Culprit lesion was identified, and its characteristics were detailed. Any other angiographically significant lesions (> 50% diameter stenosis in a > 2mm vessel) were evaluated and detailed. The decision to perform PCI to a non-culprit lesion in the same session, a separate future session or to leave it for medical therapy was per operator discretion. Relevant angiographic parameters including initial and final Thrombolysis In Myocardial Infarction (TIMI) flow, TIMI thrombus grade, any procedural complications, besides the number, locations and features of non-culprit lesions, were assessed and tabulated. All patients were initiated on guidelines-directed medical therapy (GDMT) for STEMI and relevant comorbidities before discharge.

The mean follow-up duration in our study was 14 ± 1.8 months. During the follow-up, patients were periodically assessed for the occurrence of any MACE, recurrent acute coronary syndrome (ACS) or heart failure hospitalizations. Other adverse events during the study period such as exercise intolerance impacting the quality of life, heart failure emergency visits, repeated hospitalization, stent failure or urgent unplanned revascularization.

### Statistical analysis

Statistical package for social science (SPSS) software, version 22 for Microsoft Windows (SPSS Inc., Chicago, IL, USA) was used for data analysis. Categorical data were presented as frequency and percentages (*n* (%)) and correlations among them were analyzed by Chi-square test or Fisher's exact test as appropriate. Continuous data were subjected to normality testing using Shapiro–wilk test and (if needed) visual assessment of histogram plots; and were presented as mean ± (standard deviation) or median [interquartile range], then were compared using independent samples t-test or Mann–Whitney test as appropriate. Regression analyses were conducted to identify statistically significant predictors of outcome endpoints. A probability *p*-value ≤ 0.05 was considered statistically significant.

## Results

In this prospective study, a total of 492 STEMI patients presented to National Heart Institute and Cairo University Hospitals for pPCI. Among the study cohort, 98 patients (20%) were aged ≤ 45 years and comprised the young STEMI group. The baseline demographic, risk factors, laboratory, echocardiographic and interventional data of the young STEMI patients contrasted to those aged > 45 years (controls) are demonstrated in Tables 1 and 2.

**Table 1:** Baseline demographic, risk factors and laboratory data of both study groups

	Age group		*p*-value*
	≤ 45 years (*n* = 98)	> 45 years (*n* = 394)	
Male gender	85 (86.7%)	286 (72.6%)	** 0.004**
BMI (kg/m^2^)	27.51 ± 3.6	28.58 ± 3.8	** 0.011**
Risk factors			
DM	20 (20.4%)	175 (44.7%)	** < 0.001**
HTN	20 (20.4%)	177 (44.9%)	** < 0.001**
Dyslipidaemia	12 (12.7%)	86 (21.8%)	**0.038**
Smoker	71 (72.4%)	196 (49.7%)	** < 0.001**
FH	13 (13.3%)	19 (4.8%)	**0.002**
Hb (g/dl)	13.98 ± 1.8	13.29 ± 1.5	** 0.001**
Serum creatinine (mg/dl)	0.97 ± 0.3	1.17 ± 0.5	** < 0.001**
e-GFR (ml/min/1.73 m^2^)	98.58 ± 7.8	74.09 ± 6.9	** < 0.001**

Bold values statistical significance, when *p* value is <0.05

Continuous variables are expressed as mean ± standard deviation, categorical variables are expressed as numbers (percentage). *Chi-square test was used to compare the frequency differences between groups, while T-tests was used to compare the mean differences between groups

BMI = Body mass index, DM = Diabetes mellitus, e-GFR = Estimated glomerular filtration rate, Hb = Hemoglobin, FH = Family history of premature coronary artery disease, HTN = Hypertension

**Table 2:** Baseline echocardiographic and interventional data of both study groups

	Age group		*p*-value
	≤ 45 years (*n* = 98)	> 45 years (*n* = 394)	
EF%	52.50 ± 8.4	47.69 ± 8.5	** < 0.001**
RWMA Score	19.45 ± 3.1	21.67 ± 3.7	** < 0.001**
WMS index	1.20 ± 0.2	1.35 ± 0.2	** < 0.001**
Culprit lesion			
LAD	65 (66.3%)	263 (66.7%)	0.942
LCX	10 (10.2%)	44 (11.2%)	
RCA	23 (23.5%)	87 (22.1%)	
Successful revascularization	93 (94.9%)	377 (95.7%)	0.736
Large thrombus burden	25 (25.5%)	83 (21.1%)	0.342
Scheduled for CABG	1 (1%)	20 (5.1%)	**0.046**

Bold values statistical significance, when *p* value is <0.05

EF = Ejection fraction, LAD = Left anterior descending coronary artery, LCX = Left circumflex artery, RAC = Right coronary artery, RWMA = Regional wall motion abnormality, WMS = Wall motion score

Younger STEMI patients showed better LV ejection fraction, lesser wall motion abnormality score and wall motion score index compared to older STEMI patients. Only 1 patient (1%) in the ≤ 45 years compared to 20 patients (5.1%) in the > 45 years group were found to have multiple severe non-culprit lesions, therefore, received balloon and/or thrombus aspiration only for the culprit lesion then scheduled for coronary artery bypass graft (CABG) for complete surgical revascularization.

### Age-related impact on adverse clinical outcomes through the follow-up

Encountering adverse clinical outcomes during the period of follow-up was associated with increasing age. Mean age of those who had any adverse event was significantly older than those with event-free follow-up periods, (57.7 ± 10.4 years vs. 53.9 ± 11.3 years, *p* < 0.001).

Evaluating rates of adverse events according to the age group showed a generally lower event rate in ≤ 45 years STEMI patients compared to those > 45 years and is demonstrated in Table 3. Heart failure requiring hospitalization or urgent hospital visit occurred in 18 (18.4%) vs. 137 (34.8%) in the younger compared to the older group, *p* = 0.002. MACE defined as a composite of death, non-fatal MI or TLR occurred in 10 (10.2%) in the ≤ 45 years compared to 94 (23.9%) in the > 45 years STEMI patients, *p* = 0.003. Death from any cause was comparable and occurred in 3 (3.1%) and 16 (4.1%) in those ≤ 45 years and > 45 years, respectively, *p* = 0.646. Worth mentioning that all death events in this study occurred within the first 60-days after the index event. Also, all CABG procedures (1% and 5% in those ≤ 45 and > 45 years, respectively) were planned procedures, not due to new ischemia, thereby, were not counted as MACE or adverse events. Subjective feeling of effort intolerance impairing quality of life but without objective evidence of heart failure (signs, imaging or laboratory) was met in 28 (28.6%) vs. 170 (43.1%) in the younger compared to the older STEMI groups, *p* = 0.008.

**Table 3:** Relationship between adverse clinical outcomes and age

	Age group		*p*-value
	≤ 45 years (*n* = 98)	> 45 years (*n* = 394)	
Heart failure	18 (18.4%)	137 (34.8%)	**0.002**
Repeated hospital admission	7 (7.1%)	45 (11.4%)	0.218
Stent failure	2 (2%)	3 (0.8%)	0.204
New revascularization	7 (7.1%)	47 (11.9%)	0.175
Stroke	1 (1%)	5 (1.3%)	0.657
Recurrent MI	5 (5.1%)	16 (4.1%)	0.648
Death	3 (3.1%)	16 (4.1%)	0.646
MACE	10 (10.2%)	94 (23.9%)	**0.003**
Any adverse outcome	37 (37.8%)	225 (57.1%)	**0.001**
Effort intolerance	28 (28.6%)	170 (43.1%)	**0.008**

Bold values statistical significance, when *p* value is <0.05

MACE = Major adverse cardiovascular events (defined as all cause death; MI or target vessel revascularization), MI = Myocardial infarction

### Predictors of adverse outcomes

In the whole study group (*n* = 492), age > 45 years, diabetes mellitus, higher serum creatinine, higher baseline serum creatinine, lower predischarge ejection fraction, larger LV end-diastolic volumes and higher WMS index were predictors for adverse outcomes through the follow-up period. Conversely, looking specifically at the young STEMI patients (*n* = 98), hypertension, dyslipidemia, family history or premature CAD and lower eGFR levels were significant predictors for the occurrence of adverse outcomes through the follow-up period. Because post-pPCI heart failure was the most prevalent and problematic consequence, its predictors were specifically sought to define related independent predictors. In multivariate regression models, age > 45 years, hypertension, lower eGFR, and higher WMS index in the predischarge echo were identified as independent predictors for post-pPCI heart failure. These data are illustrated in Table 4.

**Table 4:** Predictors of adverse events and post-pPCI heart failure

Predictor	OR (95% CI)	*p*-value
**Predictors of adverse events in the whole study group**		
Age group (> 45 years)	2.757 (1.378–5.519)	**0.004**
Male sex	0.895 (0.539–1.487)	0.670
Diabetes mellitus	1.801 (1.164–2.785)	**0.008**
Serum creatinine	2.132 (1.191–3.817)	**0.011**
EF	0.934 (0.901–0.970)	** < 0.001**
LVEDD	1.061 (1.008–1.117)	**0.025**
WMS Index	12.686 (4.843–23.226)	**0.005**
**Predictors of adverse events in young STEMI patients (< 45 years)**		
Male sex	1.048 (0.416–2.159)	0.898
Hypertension	2.794 (1.165–6.698)	**0.021**
Dyslipidemia	3.138 (1.102–8.941)	**0.032**
FH of p-CAD	0.029 (0.004–0.205)	** < 0.001**
e-GFR	0.955 (0.936–0.975)	** < 0.001**
**Predictors of post-pPCI heart failure in the whole group**		
Age group (> 45 years)	2.096 (1.195–3.674)	**0.010**
Male sex	0.705 (0.393–1.264)	0.241
Hypertension	1.677 (1.131–2.486)	**0.010**
e-GFR	0.987 (0.975–0.998)	**0.025**
WMS index	8.766 (1.192–14.408)	**0.012**

Bold values statistical significance, when *p* value is <0.05

CI = Confidence interval, EF = Ejection fraction, eGFR = Estimated glomerular filtration rate, FH of p-CAD = Family history of premature CAD, LVEDD = left ventricular end diastolic dimension, OR = Odds ratio, WMS = Wall motion score

## Discussion

This observational cross-sectional study recruited 492 acute STEMI patients presenting for pPCI in 2 large tertiary Egyptian centers. We found that young age (≤ 45 years) is not uncommon among STEMI presentations in Egypt representing one-fifth of all comers. Male gender by far represented the majority in both age groups, but, with a significantly higher predominance in the younger- compared to the older group. Compared to older patients, young STEMI patients had significantly higher rates of smoking and family history of premature CAD, while contrarily had lower rates of other conventional CAD risk factors such as diabetes, hypertension and dyslipidemia.

Acute MI in the young has been an increasing problem that has attracted a lot of attention in the recent years. Despite the substantial improvement in public awareness, pPCI availability and techniques and antithrombotic therapies, CAD morbidity is increasing with an alarming rate [13]. The good aspect of reducing acute MI-related mortalities is actually paralleled by improved longevity, with higher rates and severity of increased morbidity and lifetime health expenditure costs. This is particularly important in low-to-mid-income countries (LMIC) where young patients are often the primary family earners, and hence, significant illness would impair not a single person ability to work, but multiple dependents, with a huge negative impact on national productivity and the public health systems.

In the large INTERHEART study that included data from 52 countries and a total of > 30,000 participants, LMIC countries had almost 80% of the global burden of CAD [8]. More specifically, the Middle-East region (with predominant LMIC) had the highest rate of acute MI patients aged < 40 years, reaching 11% compared to 3% and 4% in Western Europe and North America, respectively. Additionally, the INTERHEART investigators have demonstrated that the median age for the first MI in the Middle-East region was almost a decade younger than that in Western Europe and North America, (51 vs. 63 and 59 years, respectively) [8].

Similar results were published in the comparison of the GULF-RACE registry including > 6700 patients from 6 Arab Middle-East countries and the multinational Global Registry of Acute Coronary Events (GRACE) recruiting nearly 4500 patients from 14 countries in North and South Americas, Europe and Australia [14]. It was demonstrated that mean age of STEMI presentations in the Gulf area was 63.8 ± 14 years opposed to 54.4 ± 13 years in the GRACE population, *p* < 0.01. Furthermore, the authors highlighted that patients < 55 years in the Gulf-RACE were 46%, opposed to 23% in the GRACE population [14].

These data emphasize the regional differences in encountering STEMI at young age, which are probably attributed to interacting genetic, epigenetic or socio-behavioral factors. Nevertheless, it assures that LMIC countries as in the Middle East are in a more grave need to investigate and address such rising problem.

Regarding gender, it is established that male sex often dominates observational registries, and randomized studies concerning CAD. Conveniently, this is because of the protective effects of female sex hormones against vascular walls inflammation and atherosclerotic during the childbearing period, with later catch-up after menopause [15]. However, it should be taken into account that the socio-behavioral impacts like heavy smoking, alcohol and occupational stressors tend to be higher on the males' side, particularly in Middle-Eastern populations. Both of these postulations are particularly valid in our study, to find a male gender dominance in both age groups, yet with a significantly higher male proportion in the young < 45 years group (classically, the premenopausal age).

This was in accordance with another study from the same region (Middle East) reporting the First Jordanian Percutaneous Coronary Intervention Registry and involving 706 STEMI patients. In their report, Obeidat et al., had near domination (96%) of male gender in < 45-year-old STEMI patients compared to 82% in those > 45 years [16].

Despite this strong association, male gender in our study was not a significant predictor for adverse outcomes neither in the whole study group, nor in those ≤ 45 years. It is difficult to ascertain whether male gender predisposes to CAD but not necessarily predispose to subsequent adverse events or failure to elicit a significant predictive relationship was simply because of near dominance of male gender in our study. However, Thomas et al., have demonstrated that young male STEMI patients showed lower mortality compared to young female patients [17]. Probably, larger multicenter studies are recommended to explore this relation.

Identification of any peculiar risk factor(s) that characterize young STEMI patients could have remarkable clinical importance, particularly if modifiable. In our cohort, the main risk factor among the young STEMI patients was heavy smoking followed by family history of premature CAD and was significantly more prevalent compared to the older STEMI subgroup, (72.4 vs. 49.7% and 13.3 vs. 4.8%, respectively). Contrarily, all other traditional risk factors for CAD, such as diabetes, hypertension or dyslipidemia were significantly less prevalent among our young STEMI compared to the older STEMI patients.

Many studies conducted in the west have shown that family history of premature CAD, hypercholesterolemia, sedentary lifestyles, obesity or smoking represent a frequent predisposition in young patients with CAD [18, 19]. On the other hand, in a large study recruiting multi-ethnic Asian patients, Tung et al., demonstrated a significantly higher rates of smoking, Indian race and family history of premature CAD, opposed to significantly lower rates of diabetes, hypertension and dyslipidemia in young compared to older STEMI patients [20].

Better LV tolerability to the acute insult and limited myocardial damage in the younger STEMI patients, indicated by better LVEF and lesser wall motion abnormality scores, probably reflect the lower comorbidity burden specifically diabetes, hypertension and dyslipidemia in the younger patients. It is imperative that these comorbidities, particularly diabetes mellitus, significantly impair the coronary microvasculature and are associated with larger damage and impaired recovery after STEMI treated with pPCI [21, 22].

Exploring the angiographic features, the LAD was the most encountered culprit vessel, followed by the RCA in both age groups. There were no significant differences concerning the infarct-related artery, or thrombus burden between younger compared to older STEMI patients. Also, success and adequacy of the pPCI procedure were comparable between the 2 groups. There was a small yet significant difference in the number of patients found to have multiple severe non-culprit lesions who underwent balloon angioplasty and/or thrombus aspiration then scheduled for CABG for complete surgical revascularization, being 1% vs 5% in the young compared to the older STEMI subgroups.

Highlighting the clinical outcomes, it was plausible that increasing age was associated with more adverse events. In this study, compared to the older patients, young STEMI patients showed significantly lower rates of MACE, heart failure or encountering any adverse event through the follow-up period, nevertheless with comparable mortality rates.

In a large study that had evaluated 28,778 acute coronary syndrome patients in 76 hospitals, younger patients were significantly less likely to develop heart failure (Killip II-to-IV) compared to older patients, 5.2 vs. 23.0%, *p* < 0.001 [23]. Another recently published study has shown that among STEMI patients, in-hospital, short- and mid-term survival were significantly better in young compared with older patients. The long-term survival was also better in the young STEMI compared with older patients with few of them dying beyond 1 year of the index STEMI [20].

The astonishing comparable between-groups mortality rate in our study is possibly because of the relatively short follow-up period. However, bearing in mind that all encountered death events were relatively early, another contributing scenario might have taken place. Unfortunately, younger patients are generally more resistant to be compliant with regular therapy (e.g., antiplatelets), which might have contributed to the numerically higher rates of stent failure and recurrent MI compared to the older subgroup. Whether unreported cases of treatment discontinuation were responsible for the catch-up in rates of death with older patients is very likely. A longer period of follow-up shall be quite informative if separation of the survival curves occurred.

This study has demonstrated that age > 45 years, diabetes mellitus, higher serum creatinine and poorer LV function in predischarge echocardiogram were significant predictors for adverse clinical outcomes in STEMI all comers. Likewise, hypertension, dyslipidemia, family history of premature CAD and lower eGFR were identified as significant predictors for adverse clinical outcomes in young (≤ 45 years) STEMI patients. Similar findings were demonstrated by Pablo et al., who examined > 19,000 patients and showed that MACE in the follow-up was linked with dyslipidemia and lower LVEF [24]. Also, in another study evaluating the impact of impaired renal function on cardiovascular outcomes, the investigators have shown that impaired renal functions were associated with higher rates of acute coronary syndrome, myocardial infarction, decompensated heart failure, stroke and death [25].

## Conclusions

STEMI in young individuals is not an uncommon scenario particularly in the Middle-East region and has a huge social and national impact. Young patients (≤ 45 years) represented one fifth of STEMI all-comers in two large Egyptian referral pPCI centers. Compared to those > 45 years, young STEMI patients had significantly higher rates of heavy smoking (72%) and potential genetic predisposition (13%), while were less likely to exhibit other conventional risk factors of ASCVD, like diabetes, hypertension or dyslipidemia. Public awareness that smoking can cause STEMI in young healthy individuals is critically required in our population.

### Study limitations

This study had certain limitations. Despite recruiting all eligible STEMI patients from 2 large pPCI Egyptian centers, the sample size is considered relatively small. This is probably attributed to the selective recruitment, with several exclusion criteria mentioned in the study protocol. Also, the partial overlap of the study recruitment period with COVID-19 peaking (last 3-months of recruitment period), could have impacted STEMI presentations, with declining numbers and potentially affecting patients’ profiles [26]. The exclusion of patients with coma, cardiogenic shock, or severe renal impairment would hamper generalization of the study results to these subgroups, however, there is established evidence that these subgroups have characteristic course and outcomes different from the general cohort, requiring to be assessed in dedicated studies. Another limitation may be related to having the recruitment from 2 Egyptian centers in the capital, Cairo, which would affect the generalizability of the conclusions to other rural areas with often lower rates of smoking. Nevertheless, efforts to raise public awareness against smoking, particularly among the youth would benefit anyways.

## Data Availability

Data can be provided (anonymized) upon reasonable request from the corresponding author.

## Abbreviations

CAD: Coronary artery disease
ACS: Acute coronary syndrome
AMI: Acute myocardial infarction
ASCVD: Atherosclerotic cardiovascular disease
CABG: Coronary artery bypass grafting
CAD: Coronary artery disease
COVID-19: Coronary virus disease 2019
eGFR: Estimated glomerular filtration rate
GDMT: Guidelines-directed medical therapy
LAD: Left anterior descending
LCx: Left circumflex
LMIC: Low-to-mid-income countries
LVEF: Left ventricular ejection fraction
MACE: Major adverse cardiovascular events
MDRD: Modification of diet in renal disease
MENA: Middle East and North Africa
MI: Myocardial infarction
pPCI: Primary percutaneous coronary intervention
RCA: Right coronary artery
RWMS: Regional wall motion score
STEMI: ST-segment elevation myocardial infarction
TIMI: Thrombolysis in myocardial infarction
TLR: Target lesion revascularization
WMSI: Wall motion score index
